# Nerve entrapment syndromes of the lower limb: a pictorial review

**DOI:** 10.1186/s13244-023-01514-6

**Published:** 2023-10-02

**Authors:** Shanesh Kumar, Mohammad Danish Mangi, Steven Zadow, WanYin Lim

**Affiliations:** 1 Department of Radiology, Royal Adelaide Hospital, Port Rd, Adelaide, Australia; 2 Adelaide Medical School, The University of Adelaide, Adelaide, Australia; 3 Department of Medical Imaging, Flinders Medical Centre, Flinders Drive, Bedford Park, Australia; 4 Jones Radiology, Eastwood, Australia

**Keywords:** Nerve compression syndromes, Leg, Ultrasonography, Magnetic resonance imaging

## Abstract

Peripheral nerves of the lower limb may become entrapped at various points during their anatomical course. While clinical assessment and nerve conduction studies are the mainstay of diagnosis, there are multiple imaging options, specifically ultrasound and magnetic resonance imaging (MRI), which offer important information about the potential cause and location of nerve entrapment that can help guide management. This article overviews the anatomical course of various lower limb nerves, including the sciatic nerve, tibial nerve, medial plantar nerve, lateral plantar nerve, digital nerves, common peroneal nerve, deep peroneal nerve, superficial peroneal nerve, sural nerve, obturator nerve, lateral femoral cutaneous nerve and femoral nerve. The common locations and causes of entrapments for each of the nerves are explained. Common ultrasound and MRI findings of nerve entrapments, direct and indirect, are described, and various examples of the more commonly observed cases of lower limb nerve entrapments are provided.

**Critical relevance statement** This article describes the common sites of lower limb nerve entrapments and their imaging features. It equips radiologists with the knowledge needed to approach the assessment of entrapment neuropathies, which are a critically important cause of pain and functional impairment.

**Key points**

• Ultrasound and MRI are commonly used to investigate nerve entrapment syndromes.

• Ultrasound findings include nerve hypo-echogenicity, calibre changes and the sonographic Tinel’s sign.

• MRI findings include increased nerve T2 signal, muscle atrophy and denervation oedema.

• Imaging can reveal causative lesions, including scarring, masses and anatomical variants.

**Graphical Abstract:**

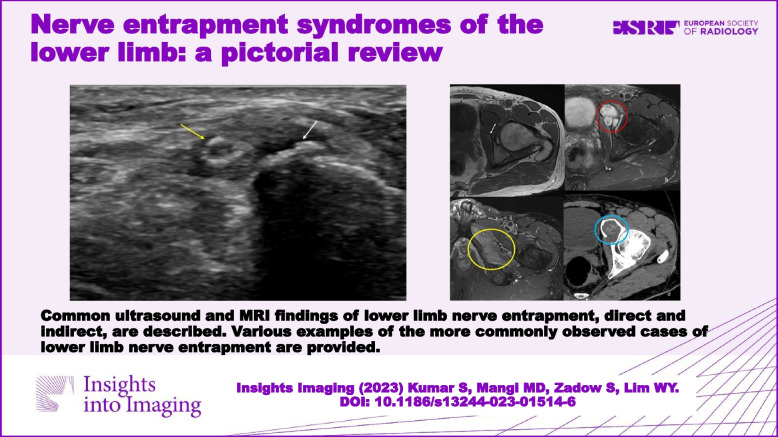

## Introduction

Peripheral neuropathies are an important cause of pain and functional impairment [1–3]. They can be categorised into compressive and non-compressive causes. Compression of peripheral nerves can occur at specific locations as the nerve passes through a fibro-osseous tunnel or an opening in a fibrous or muscular structure [4–6]. Non-compressive aetiologies include traumatic, infectious and inflammatory conditions [7]. Traditionally, the diagnosis was obtained from clinical history, physical examination and electrophysiological evaluation [8, 9]. However, this is complicated by the fact that the anatomy of peripheral nerves can vary markedly and the clinical presentation can be ambiguous [2]. Magnetic resonance imaging (MRI) and high-resolution ultrasonography allow direct anatomic visualisation of nerves, identification of causes of entrapment and evaluation of intrinsic abnormalities within the nerves [2, 6, 10].

Along the short axis, nerves have a honeycomb appearance, produced by hyperechoic connective tissue, called epineurium, surrounding hypoechoic nerve fascicles [11]. Along the long axis, nerves have a hypoechoic and coarse appearance [11, 12]. In a normal nerve, the fascicular pattern can be distinguished. Nerves often travel alongside vessels and, hence, identifying nearby vessels can be a handy tool for locating a nerve of interest [13]. On MRI, nerves are normally hypointense on T1-weighted images and isointense to hyperintense on fluid-sensitive sequences [1].

Features of nerve entrapment on high-resolution ultrasound include focal nerve enlargement (increased cross-sectional area) and loss of echogenicity [13, 14]. Abnormal MRI features of peripheral nerves in entrapment include increased signal on T2-weighted imaging and increased cross-sectional area of the nerve [3]. An additional technique which is increasingly studied in MR neurography is diffusion tensor imaging (DTI) [15]. This technique allows accurate visualisation of nerves by means of their fractional anisotropy (FA) and microstructural properties [15, 16]. Pathological conditions of peripheral nerves lead to decreased FA values [15]. DTI has mostly been utilised in evaluating acute nerve injuries, inflammatory neuropathies, peripheral nerve tumours and entrapment neuropathies, particularly carpal tunnel syndrome [16, 17]. However, the utility of DTI in evaluating lower limb peripheral nerve entrapment has not yet been investigated [16].

The purpose of this article is to review and illustrate the imaging features of entrapment neuropathies of the lower limb.

### Overview of lower limb nerves and their entrapment syndromes

The lumbosacral plexus provides motor and sensory innervation to the pelvis and lower limb [18]. It is comprised of the lumbar plexus and the sacral plexus. The lumbar plexus is formed from the anterior rami of L1-L4 nerve roots with occasional contribution from T12. The sacral plexus is formed from the anterior rami of L4-S4 nerves. The two plexuses are connected by the lumbosacral trunk, comprised of the L4 and L5 nerve roots, to form the lumbosacral plexus [19].

### Sciatic nerve

The sciatic nerve arises from the anterior divisions of the L4 to S3 nerve roots and leaves the pelvis through the greater sciatic foramen at the inferior border of the piriformis muscle [1, 18]. It provides motor supply to the biceps femoris, semitendinosus, semimembranosus and the hamstring portion of the adductor magnus. The sciatic nerve divides into the tibial nerve and common peroneal nerve which provide motor and sensory supply to the anterior, lateral and posterior lower leg as well as to the sole of the foot. The proximal sciatic nerve may be compromised by trauma, tumours, haematomas and compression in the subgluteal space is termed deep gluteal syndrome. Causes of this include piriformis syndrome, ischiofemoral impingement and fibrovascular bands [20]. The distal branches can be damaged by fractures, bandages or tumours such as ganglion cysts [2, 3, 21].

**Fig. 1:**
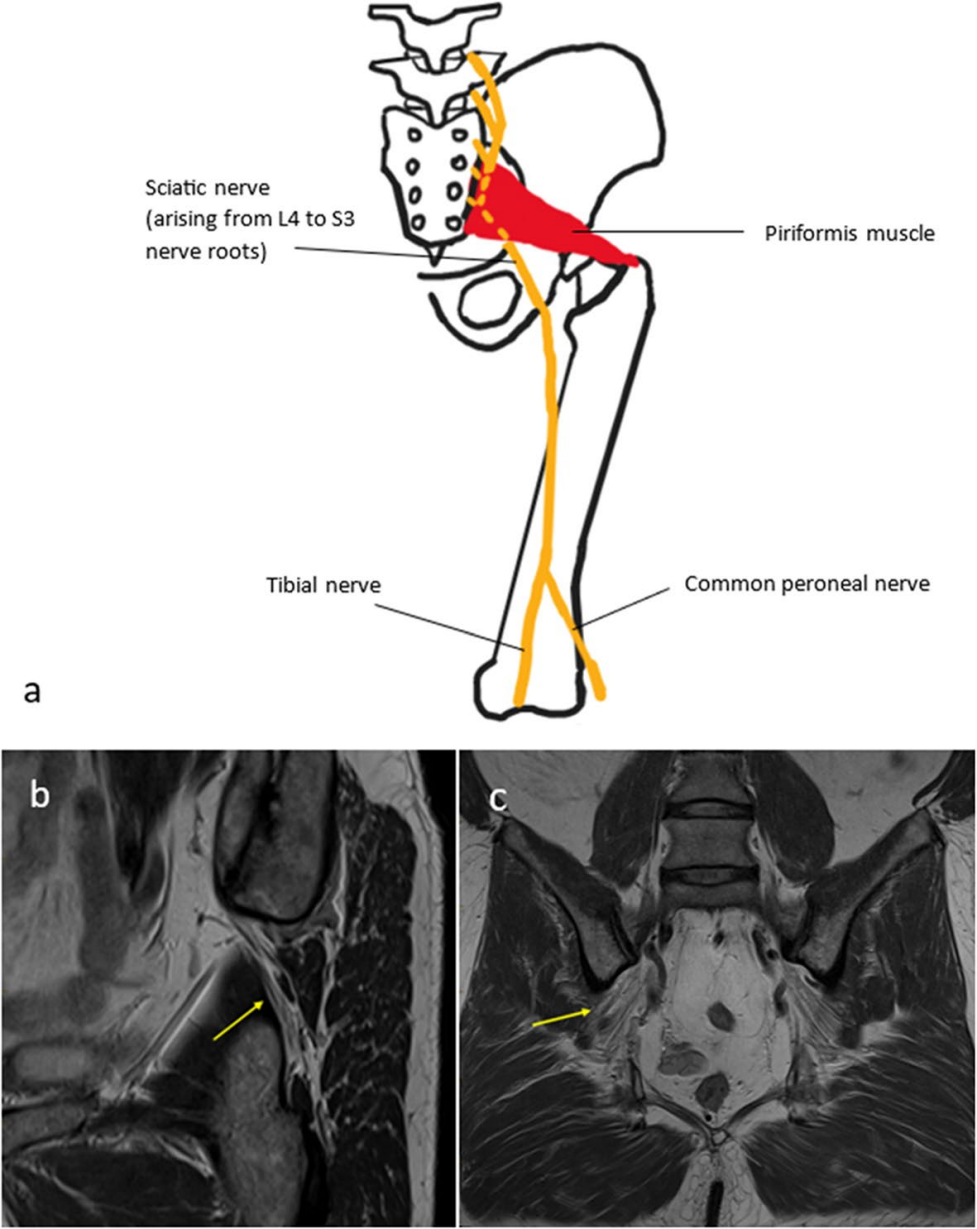
**a** The conventional relation of the sciatic nerve to the piriformis muscle is illustrated. **b**, **c** A 41-year-old male with piriformis syndrome. Sagittal oblique PD (**b**) and coronal PD (**c**) of the right hip show that the sciatic nerve (yellow arrow) has an anomalous path, with a branch piercing the right piriformis muscle

Piriformis syndrome (illustrated in Fig. 1) is a controversial diagnosis, with no consensus about diagnostic criteria. Hence, the diagnosis is typically one of exclusion [14, 22, 23]. In particular, it is important to exclude lumbar spinal pathology before making the diagnosis of piriformis syndrome [24]. The aetiology of this syndrome is nerve compression or irritation in the greater sciatic foramen by disorders related to the piriformis muscle [1, 8, 14, 24]. This can be primary, as a result of intrinsic piriformis muscle pathology such as hypertrophy. More commonly, it is secondary to other pathology at the level of the greater sciatic foramen, for example space-occupying lesions or oedema from sacroiliitis [24]. In a minority of patients, the sciatic nerve passes through the piriformis muscle. Patients suffering from this condition present with neuropathic pain in the sciatic nerve distribution, i.e. the leg and buttock [8]. MRI can demonstrate anatomical variation of the sciatic nerve and hypertrophy of the piriformis muscle. It can also reveal secondary causes such as mass lesions. Electromyography can aid in diagnosis [22]. Treatment for piriformis syndrome includes physical therapy, and a combination of local anaesthetic and corticosteroid injections, which may also have a diagnostic role [16].

### Tibial nerve

The tibial nerve arises from the sciatic nerve and descends into the posterior compartment of the lower leg deep to the soleus, plantaris, and gastrocnemius muscles. It then enters the tarsal tunnel, which is formed by the calcaneus, talus, medial malleolus and flexor retinaculum—entrapment is common at this site and is termed “tarsal tunnel syndrome”. The tibial nerve is accompanied in the tarsal tunnel by the tibialis posterior, flexor digitorum longus and flexor hallucis longus tendons, and the posterior tibial vein and artery. The tibial nerve then divides into the medial calcaneal nerve and the lateral and medial plantar nerves at the distal tarsal tunnel [1, 18]. The tibial nerve provides sensory supply to the posterolateral leg and, via the medial and lateral plantar nerves, to the sole of the foot. It provides motor supply to the posterior compartment of the leg and, via the medial and lateral plantar nerves, supplies most intrinsic muscles of the foot.

**Fig. 2:**
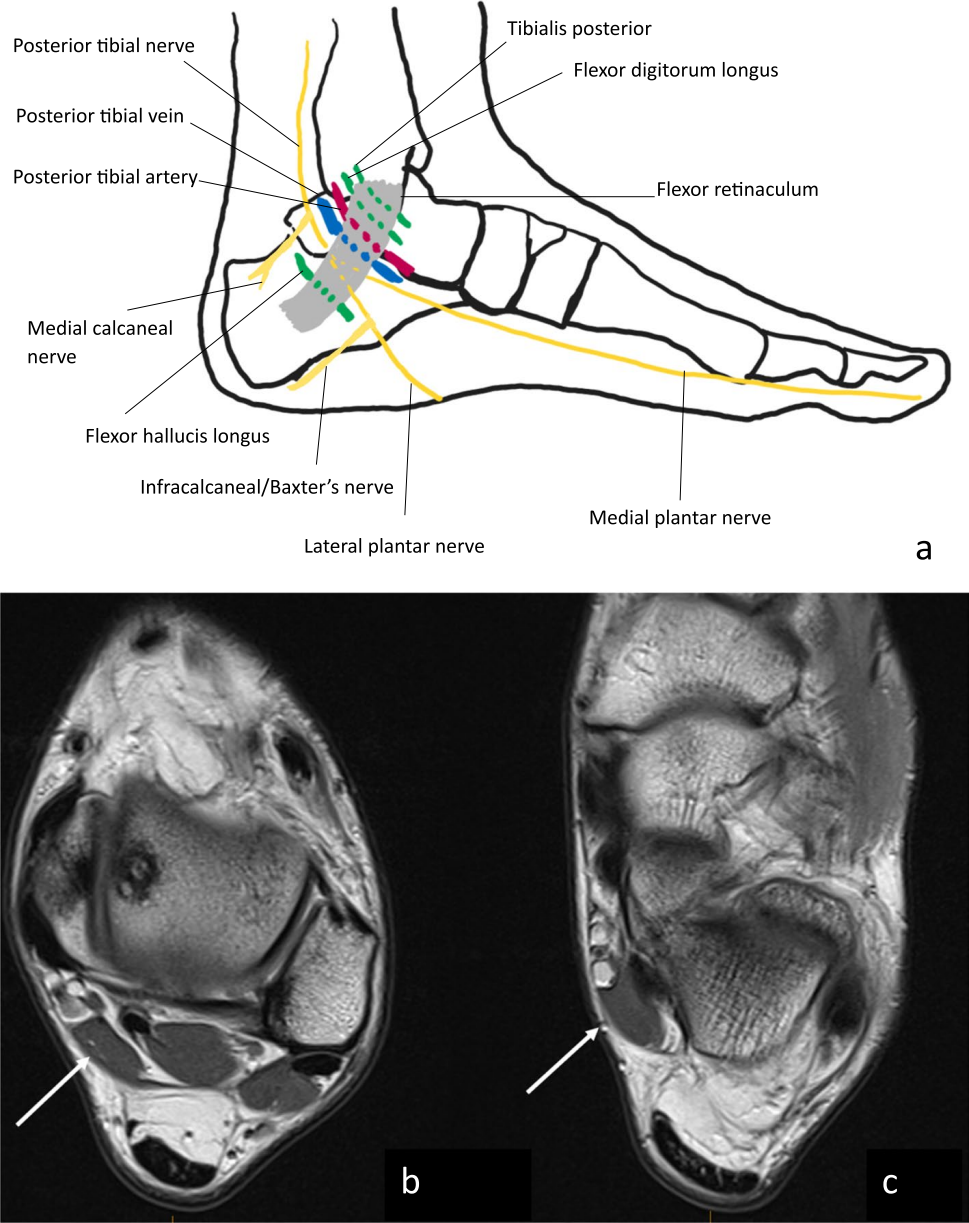
**a** The contents of the tarsal tunnel are illustrated. **b**, **c** An accessory muscle which can predispose to tarsal tunnel syndrome. This case is of a 20-year-old male with an accessory flexor hallucis longus muscle (white arrow) within the tarsal tunnel demonstrated on sequential axial PD images

Common aetiologies of tarsal tunnel syndrome include post-traumatic fibrosis from previous fracture, tenosynovitis, external compression from space-occupying lesions such as ganglion cysts and tumours including schwannoma, dilated tortuous veins and accessory muscles. Figure 2 illustrates the contents of the tarsal tunnel and shows an example of anatomical variation of the flexor hallucis longus muscle which can predispose to tarsal tunnel syndrome. Foot deformities have also been implicated, including hindfoot valgus and less commonly forefoot pronation, pes planus and tarsal coalition [2, 25]. In up to 40% of patients, an underlying cause is not identified [2]. Compression of the proximal tibial nerve, the so-called soleal sling syndrome, is uncommon. It occurs when the proximal tibial nerve travels beneath the tendinous sling at the origin of the soleus muscle [26, 27]. Patients most commonly report burning pain and paraesthesia along the plantar aspect of the foot and digits that worsens with activity. Clinical examination can reveal sensory loss along the plantar aspect of the foot and a positive Tinel’s sign at the tarsal tunnel [25]. MRI allows the identification of space-occupying lesions, fibrotic scar tissue, anomalous muscles and hindfoot deformities [2]. Conservative treatment options include analgesia and local corticosteroid injections. Surgical decompression of the nerve is typically performed in cases of failure of conservative treatment [25, 28].

### Medial plantar nerve

**Fig. 3:**
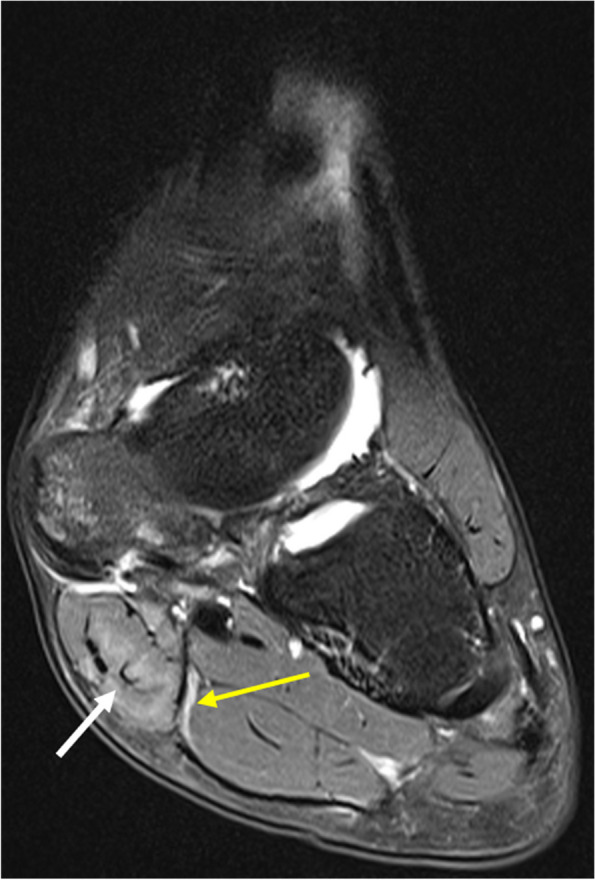
16-year-old female with medial midfoot arch pain exacerbated by running. Coronal PDFS demonstrates a hyperintense thickened medial plantar nerve (yellow arrow) just lateral to the abductor hallucis muscle (white arrow), which also demonstrates increased signal from denervation oedema. These findings are supportive of medial plantar nerve neuropathy (Jogger’s foot)

The medial plantar nerve arises from the tibial nerve at the tarsal tunnel. It crosses to the midfoot at the level of the talonavicular joint through the deep fascia of the abductor hallucis muscle to enter the medial plantar tunnel [18]. It provides sensory supply to the plantar surface of the medial three-and-a-half digits and motor supply to abductor hallucis, flexor digitorum brevis, first lumbrical and flexor hallucis brevis. Entrapment can occur at the tarsal tunnel or distal to it (depending on where it originates) or at the level of the knot of Henry (the crossing point of the flexor hallucis longus and flexor digitorum longus tendons), which is between the navicular tuberosity superiorly and the abductor hallucis muscle belly inferiorly. Entrapment at the level of the knot of Henry commonly occurs in runners and is called “Jogger’s Foot” (Fig. 3) [2, 29, 30]. Other causes include extrinsic compression from space-occupying lesions, tenosynovitis of adjacent tendons, and structural foot abnormalities [30]. Patients typically present with heel and arch pain. A positive Tinel’s sign can be elicited over the medial arch just posterior to the navicular tuberosity [30]. Entrapment or trauma affecting the medial plantar nerve’s branch to the great toe leads to benign enlargement and perineurial fibrosis, known as Joplin’s neuroma [31]. Ultrasound can show tenosynovitis, bursitis, space-occupying lesions, and nerve injuries [30]. MRI allows anatomical delineation and better detection of space-occupying lesions. Tendon sheath effusion at the knot of Henry raises the possibility of entrapment of the medial plantar nerve [2]. Denervation oedema or fatty atrophy may be seen in the muscles supplied by the nerve [2, 30]. Conservative management includes the use of orthotics, corticosteroid and local anaesthetic injections. Surgical intervention is generally reserved for patients for whom conservative management fails [18, 30].

### Lateral plantar nerve

**Fig. 4:**
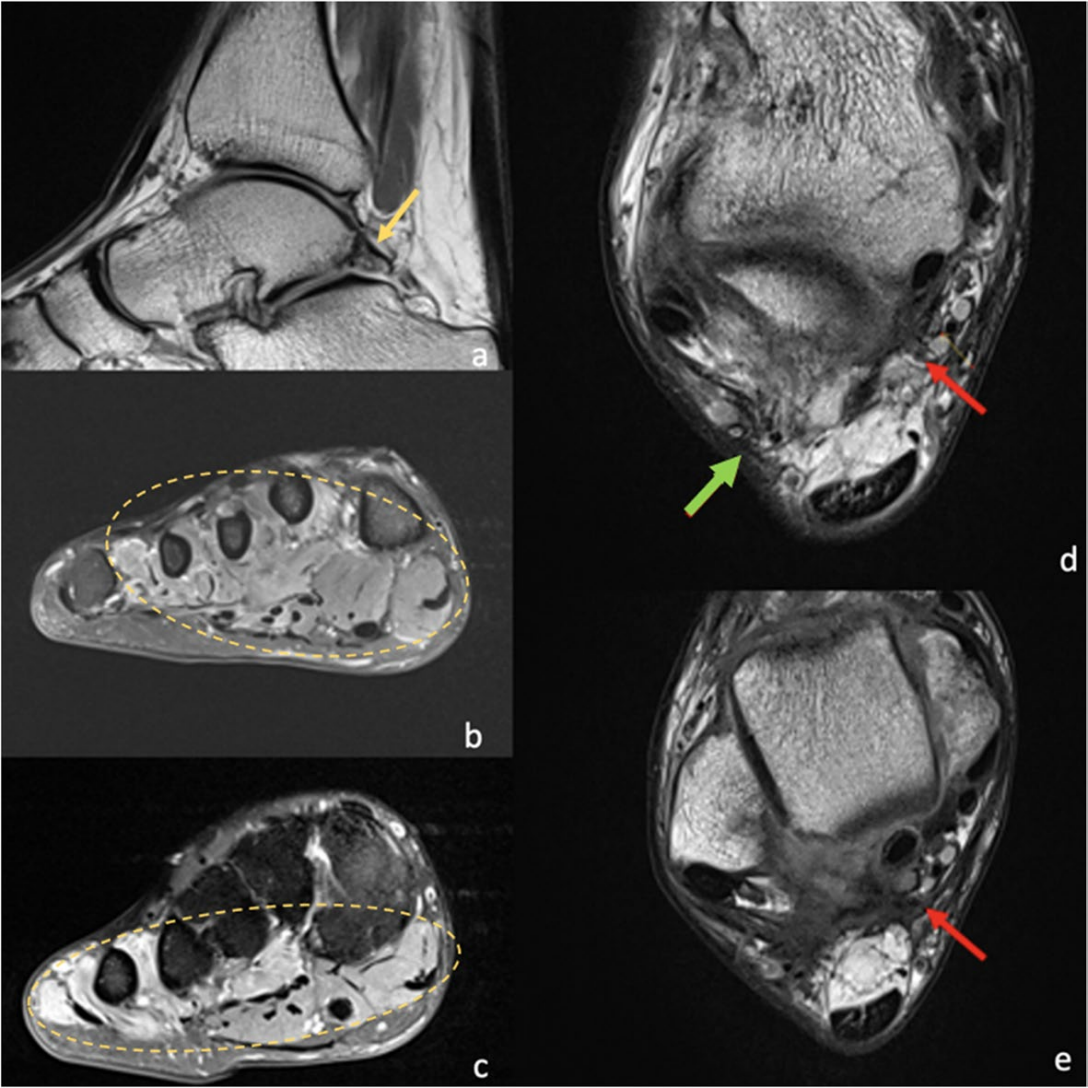
24-year-old male with posterior impingement secondary to an os trigonum, who underwent os trigonum resection. Preoperative sagittal T1 (**a**) demonstrates the prominent os trigonum (yellow arrow). Post-surgical MRI was performed due to altered sensation in the plantar forefoot laterally. Post-surgical MRI in the short axis PDFS (**b** and **c**) sequences demonstrates extensive muscle oedema and atrophy (circled) involving the lateral lumbricals, abductor digiti minimi, quadratus plantae, adductor hallucis and flexor digiti minimi brevis consistent with lateral plantar nerve impingement. Axial PD (**d** and **e**) sequences demonstrate adhesions and granulation tissue formation, extending towards the sural nerve posterolaterally (green arrow) and lateral plantar nerve medially (red arrows), resulting in impingement of the nerves

The lateral plantar nerve arises from the tibial nerve in the distal tarsal tunnel. It provides sensory supply to the lateral one-and-a-half digits and motor supply to the abductor digiti minimi, flexor digiti minimi brevis, second to fourth lumbricals, adductor hallucis, quadratus plantae and the dorsal and plantar interossei. Entrapment can occur at the tarsal tunnel or distal to it [32]. The causes of entrapment include space-occupying lesions, tenosynovitis and fibrosis of the tarsal tunnel from prior injury [32]. The most common symptoms include paraesthesia and numbness of the lateral one-third of the plantar aspect of the foot. Abductor digiti minimi weakness can be present but is often difficult to detect clinically [2, 32]. MRI allows the identification of space-occupying lesions, such as the os trigonum (Fig. 4). Denervation oedema of the intrinsic foot musculature can be visualised [2, 33].

**Fig. 5:**
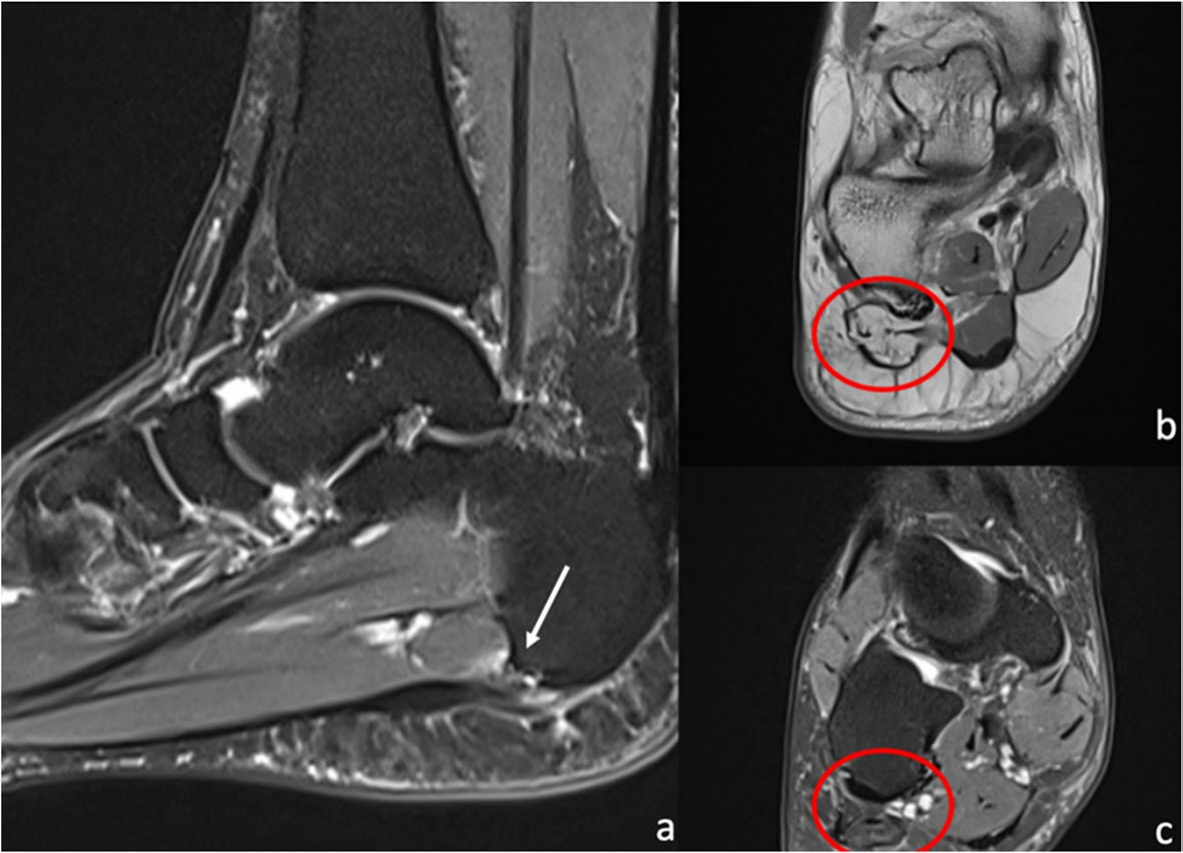
50-year-old female with acute on chronic heel pain post jogging. There is background plantar fasciopathy with nodular thickening of the plantar fascia and a high grade plantar fascial tear at the calcaneal attachment seen on the sagittal PDFS sequence (**a**, white arrow). Coronal PD (**b**) and PDFS (**c**) sequences at the level of the midfoot show severe fatty infiltration and atrophy of the abductor digiti minimi (circled), in keeping with chronic Baxter’s neuropathy

A clinically important branch of the lateral plantar nerve is the first branch, the inferior calcaneal nerve, also known as Baxter’s nerve (shown in Fig. 2(a)). Entrapment of this nerve can occur at three sites: deep to or adjacent to the fascial edge of a hypertrophied abductor hallucis muscle in long-distance runners, at the medial edge of the quadratus plantae muscle where the nerve changes from a vertical to a horizontal course, and most commonly at the medial calcaneal tuberosity [2, 28]. Entrapment at the latter site can be related to spur formation and soft-tissue inflammatory changes of plantar fasciitis (Fig. 5) [2]. Patients typically present with heel pain. MRI findings include denervation oedema or fatty atrophy of the abductor digiti minimi muscle. Abductor hallucis muscle hypertrophy and plantar fasciitis may be found as potential sources of inferior calcaneal nerve entrapment [34]. Ultrasound can be utilised to trace the nerve, to detect abductor digiti minimi atrophy and also to perform the ultrasonographic Tinel’s sign [35].

### Digital nerves

**Fig. 6:**
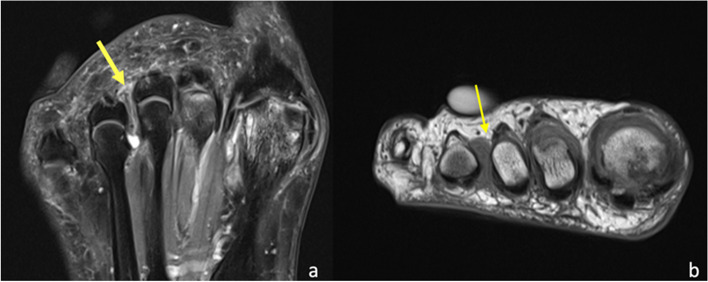
67-year-old female with forefoot pain. Coronal PDFS (**a**) and short axis T1 (**b**) sequences demonstrate dumbbell appearances of Morton’s neuroma or bursa-neuroma complex in the 3rd intermetatarsal space (yellow arrow). This corresponds to the site of the symptoms, as indicated by the patient-placed symptoms marker

The digital nerves of the foot are sensory branches given off by the medial and lateral plantar nerves. The medial plantar nerve terminates with the digital hallux nerve and the common digital nerves for the second, third, and medial fourth digits. The lateral plantar nerve provides the digital nerve for the fifth digit and the common digital nerve for the fourth and fifth digits. The digital nerves travel in narrow fibro-osseous tunnels formed by the metatarsal heads and intermetatarsal ligaments [18]. Entrapment of the digital nerves can occur in the intermetatarsal space between the metatarsal heads, plantar to the intermetatarsal ligaments [10]. The entrapment leads to perineural fibrosis of the digital nerve, a condition known as Morton’s neuroma, which is non-neoplastic (Fig. 6) [36]. The most common nerves that are affected are the second and third common digital nerves; this is thought to be due to the second and third intermetatarsal spaces being narrower compared to the first and fourth spaces [37]. Additionally, a theory suggests that the third intermetatarsal space is the most affected by Morton's neuroma because the third digital nerve is thicker due to the contribution of both medial and lateral plantar nerves [38]. The presence of hallux valgus deformity, longer second and third metatarsals, equinus contracture, tight calf muscles or wearing high-heeled shoes can shift the body weight to the region of metatarsal heads, predisposing to interdigital neuropathy [39]. More than one lesion may be detected in the same foot, and bilateral involvement can occur [36]. Classically, patients describe localised pain on the plantar surface of the foot, at the level of the metatarsal heads. The pain can radiate to the corresponding digits and there may be associated paraesthesia [40]. On examination, characteristic findings include intermetatarsal space tenderness upon compression and percussion, and a palpable Mulder’s click [41]. The differential diagnoses of Morton’s neuroma include stress fractures, Freiberg’s infraction, synovitis, bursitis, plantar plate tears and pseudoneuroma [18]. On MRI, Morton’s neuromas are isointense to skeletal muscle and well-demarcated from the surrounding hyperintense fat tissue on T1-weighted sequences. On T2-weighted sequences, they demonstrate low to intermediate signal [36]. Post-contrast sequences demonstrate solid enhancement [36].

### Common peroneal nerve

The common peroneal nerve arises from the sciatic nerve at the upper superior aspect of the popliteal fossa. It travels distally posteromedial to the short head of the biceps femoris and superficial to the lateral head of the gastrocnemius muscle. The nerve then wraps around the fibular neck before entering the peroneal tunnel between the peroneus longus and the fibula, where it divides into the superficial and deep peroneal nerves. It supplies the short head of biceps femoris, the lateral compartment of the leg via the superficial peroneal nerve and the anterior compartment of the leg via the deep peroneal nerve. In terms of sensory functions, it provides sensation to the anterolateral region of the leg and dorsum of the foot via the superficial and deep peroneal nerves. It also contributes to the lateral sural cutaneous nerve supplying sensation to the upper lateral leg and the sural nerve supplying the lower posterolateral leg [2, 42].

**Fig. 7:**
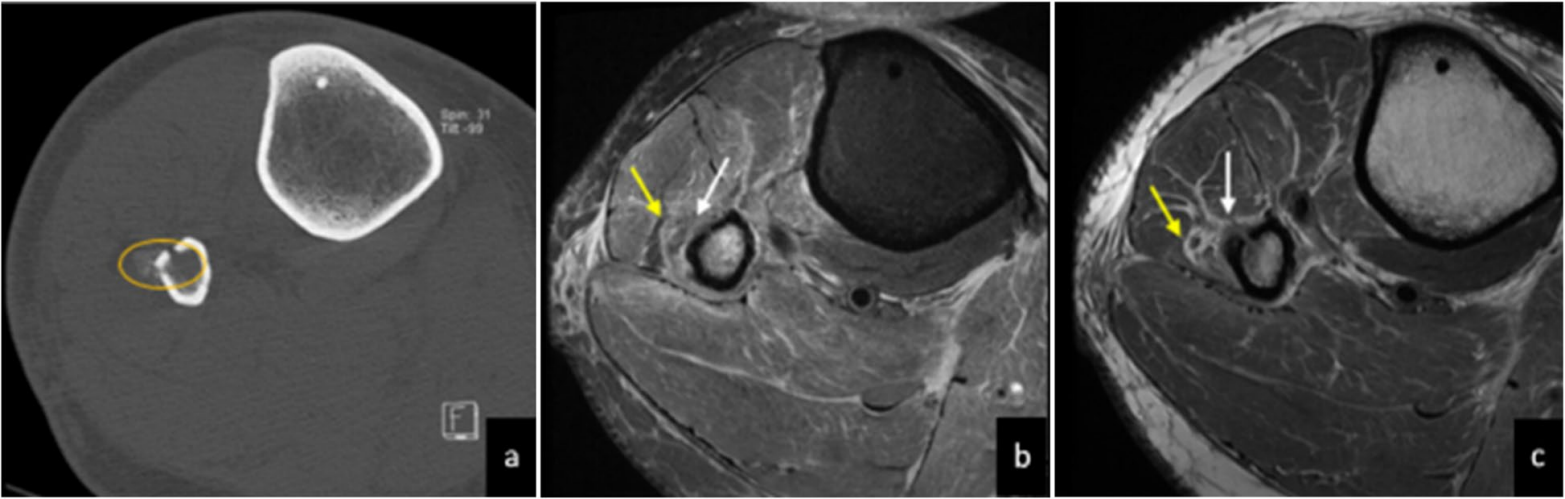
40-year-old man who had a skiing accident and sustained a high fibular fracture and syndesmotic injury. Soon after the injury, he presented with foot drop and symptoms of common peroneal nerve entrapment. CT at the level of the proximal fibula/calf shows periosteal new bone formation at site of the proximal fibular fracture (**a**). Sequential axial PDFS (**b**) and axial PD (**c** and **d**) sequences show the proximal superficial nerve (yellow arrows) and deep peroneal nerve (white arrows) shortly after the common peroneal nerve division. The deep peroneal nerve appears oedematous on the PDFS axial sequence. The superficial peroneal nerve appears oedematous, although to a lesser extent

Common peroneal neuropathy is the most common mononeuropathy of the lower limb. The two most common sites of entrapment are as it crosses the fibular neck, given its superficial location, and as it travels deep to the peroneus longus [2]. There are many causes of entrapment, including direct compression from crush injury, extended lithotomy position during surgery, prolonged squatting, short-leg cast, ganglion cysts, synovial cysts, varicosities, tumours, hypertrophy of the short head of the biceps femoris muscle, traumatic injury following high fibula fracture (Fig. 7) and knee dislocation [2, 34, 43–46]. Patients can present with the clinical symptoms of foot drop and a slapping gait [2, 8]. MRI findings include an increase in size and T2 hyperintensity of the nerve. MRI can also identify space-occupying lesions causing mass effect, and denervation oedema in the muscles supplied by the nerve [2, 34]. Treatment options depend on the cause, and include surgical treatment for an external compressing mass, activity modification, and consideration of local corticosteroid injections [18].

### Deep peroneal nerve

The deep peroneal nerve is a terminal branch of the common peroneal nerve arising from the bifurcation of the common peroneal nerve in the lateral compartment of the leg between the proximal part of the peroneus longus muscle and the neck of the fibula. The nerve then courses anteriorly deep to the extensor digitorum longus, and onto the surface of the interosseus membrane of the leg. The nerve then descends into the anterior compartment of the leg accompanied by the anterior tibial artery. Initially, the deep peroneal nerve passes lateral to the artery but distally becomes anterior. At the ankle joint, the deep peroneal nerve travels into the dorsum of the foot through the fibro-osseous anterior tarsal tunnel (formed by the inferior extensor retinaculum superficially, medial malleolus medially, lateral malleolus laterally and talonavicular joint capsule deeply), dividing within the dorsum of the foot into its two terminal branches, the lateral terminal branch and the medial terminal branch. The lateral terminal branch runs deep to the extensor digitorum brevis muscles as it passes the ankle joint. The medial terminal branch courses through the dorsal compartment of the foot, merging with the medial branch of the superficial fibular nerve at the first interosseous space.

The deep peroneal nerve provides motor function to the muscles of the anterior compartment of the leg, including the tibialis anterior, extensor digitorum longus, peroneus tertius and extensor hallucis longus, and its lateral terminal branch innervates two of the intrinsic muscles of the foot, the extensor digitorum brevis and extensor hallucis brevis.

In terms of sensory innervation, the deep peroneal nerve, during its course in the anterior compartment of the leg, provides branches to innervate the ankle joint, its medial terminal branch gives a sensory branch to the first metatarsophalangeal joint and provides cutaneous innervation to the adjacent sides and the webbing between the first two toes, and its lateral terminal branch gives sensory innervation to the tarsal joint as well as the middle three metatarsophalangeal joints [42, 47].

**Fig. 8:**
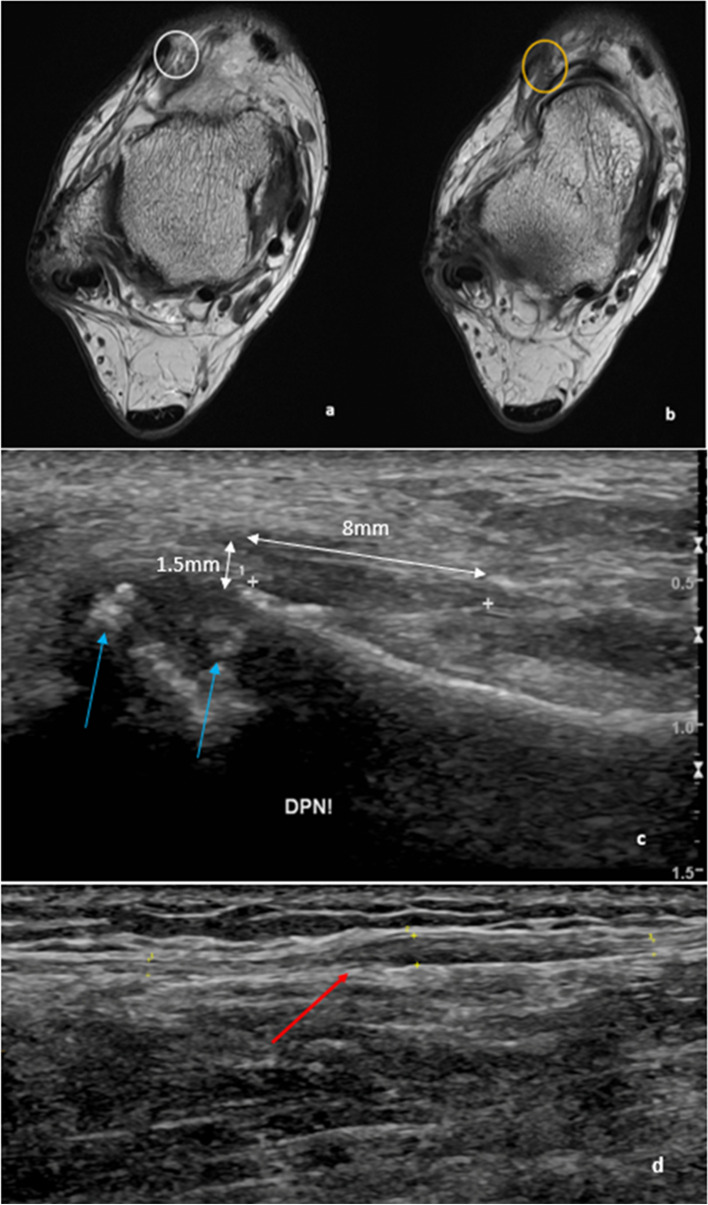
Entrapment of the deep and superficial peroneal nerves is illustrated: **a**–**c** Anterior tarsal tunnel syndrome in a 78-year-old who presented with persistent ankle and foot pain post talonavicular capsule and extensor retinacular sprain. The fat plane around the deep peroneal nerve is effaced as it courses through the anterior tarsal tunnel. Subfigures (**a**) and (**b**) are sequential axial PD slices showing the normal fat planes around the deep peroneal nerve (**a**—white circle) at proximal tunnel, before being surrounded and engulfed by the scar tissue more distally (**b**—yellow circle). On ultrasound (**c**), the longitudinal segment of the deep peroneal nerve is over 8 mm in diameter which is thickened (1.5 mm) and hypoechoic due to entrapment at the level of the anterior tarsal tunnel. Dorsal marginal osteophytes from midfoot osteoarthritis impinge on the anterior tarsal tunnel and cause deep peroneal nerve entrapment or irritation (blue arrows). **d** Targeted ultrasound of the superficial peroneal nerve. There is longitudinal thickening of the nerve (red arrow) as it passes through the crural fascia. This is 15 cm above the level of the ankle joint. This patient had a positive Tinel’s tap at the site of the nerve enlargement

Entrapment of the deep peroneal nerve can occur as it passes through the anterior tarsal tunnel, known as anterior tarsal tunnel syndrome (Fig. 8). This can be caused by extrinsic impingement by tight shoes, high heels or trauma and intrinsic impingement through dorsal osteophytes over the tibiotalar and talonavicular joints, other bony deformities or ganglion cysts. This can cause symptoms and signs including dysesthesia and paraesthesia on the dorsum of the foot, weakness or atrophy of the extensor digitorum brevis, positive Tinel sign over the course of the deep peroneal nerve and pain. The diagnosis can be supported by imaging features, such as increased nerve calibre and hypoechogenic appearance on ultrasound. MRI is particularly helpful for identifying the underlying compressive cause [47].

### Superficial peroneal nerve

The superficial peroneal nerve is a branch of the common peroneal nerve. It descends down the leg within a fascial plane between the peroneus longus and extensor digitorum longus muscles before piercing the lateral deep fascia in the lower lateral leg [48]. It provides sensation to the anterolateral leg and dorsum of the foot, except the skin between the first and second digits which is supplied by the deep peroneal nerve. It provides motor supply to the muscles of the lateral compartment of the leg. Entrapment of the nerve can occur where it pierces the fascia, for example by overstretching during inversion and plantar flexion ankle injuries which are common amongst dancers [2, 49]. Other causes include thickening of the lateral deep fascia and anatomical variations such as fascial defects with or without secondary muscle herniation [2, 18]. Space-occupying lesions, such as ganglion cysts, can also cause compressive neuropathy [50]. Patients can present with paraesthesia along the lateral aspect of the lower leg and the dorsum of the foot with sparing of the first web space and the fifth digit. A positive Tinel’s test is elicited at the entrapment site. Peroneal muscle weakness is uncommon [18]. On ultrasound, the nerve can appear thickened (Fig. 8). MRI findings include increased nerve thickness and increased T2 signal intensity. The course of the nerve can be delineated on MRI [2, 18]. Treatment options include physiotherapy, corticosteroid injection, non-steroidal anti-inflammatory drugs and surgical intervention for decompression of the nerve [49].

### Sural nerve

**Fig. 9:**
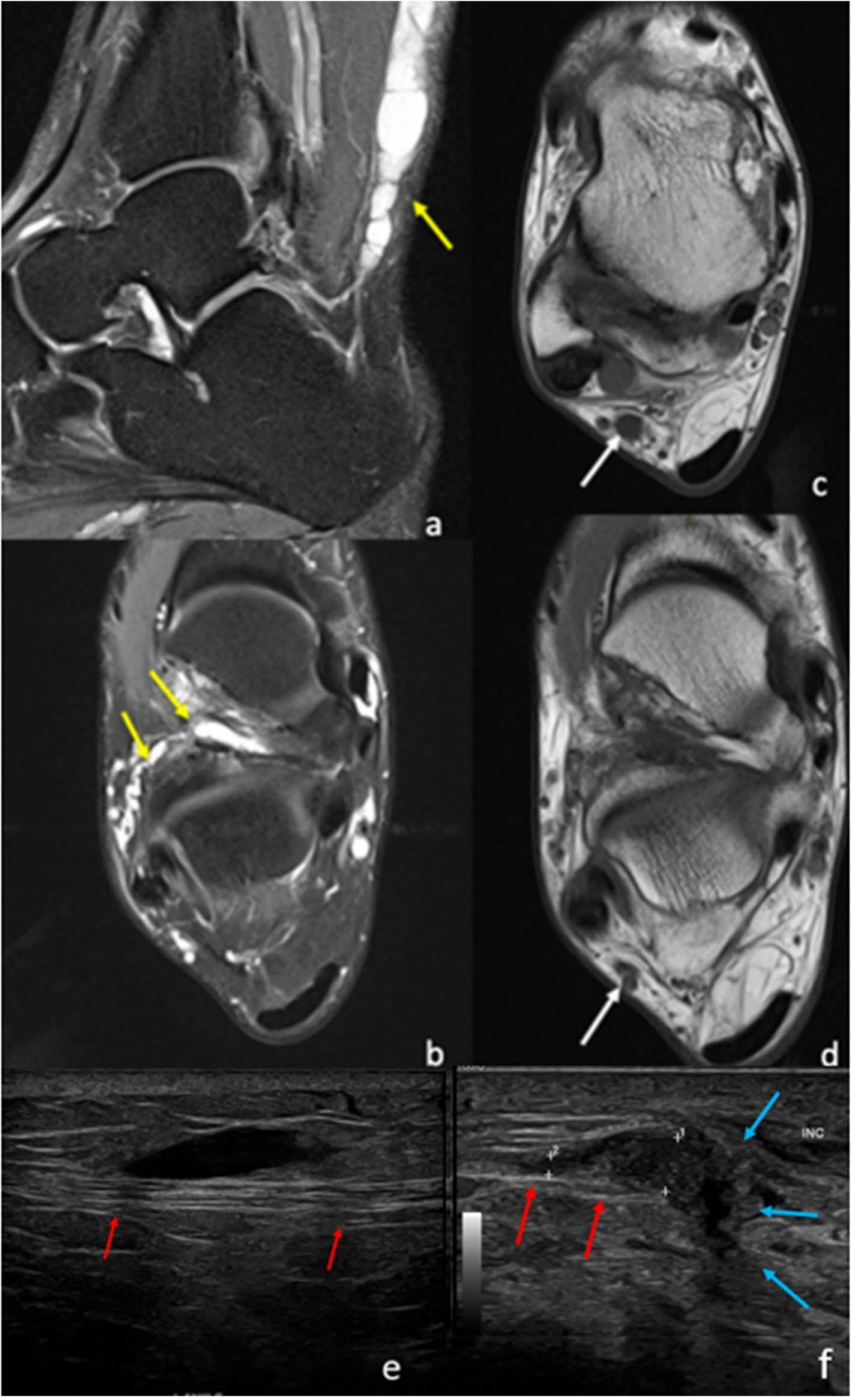
25-year-old man with sural nerve symptoms and numbness. Sagittal (**a**) and axial (**b**) PDFS and axial PD (**c** and **d**) demonstrate a ganglion (yellow arrow) arising from the sinus tarsi with superior and intraneural extension extending into the calf along the path of the sural nerve (white arrow). **e**, **f** Ultrasound images of a different patient, a 45-year-old female with neuropathic pain in the lateral foot following subtalar joint fusion from post-traumatic osteoarthritis. **e** The normal fibrillary structure of a normal segment of proximal sural nerve (red arrows). More inferiorly, at level of the surgery (**f**), there is hypoechoic enlargement of the sural nerve as it traverses the scar tissue (blue arrows), consistent with sural nerve entrapment

The sural nerve is a pure sensory nerve. It is formed by the joining of the medial sural nerve (branch of the tibial nerve) and the lateral sural cutaneous nerve (from the common peroneal nerve) at the level of the mid-leg. The nerve courses downward between the two heads of the gastrocnemius and pierces the deep fascia from within at the upper calf. More distally, it courses along the posterolateral aspect of the leg, behind the lateral malleolus and along the lateral aspect of the foot [34, 51]. The nerve supplies sensation to the lateral aspect of the ankle and foot up to the base of the fifth digit [2]. The causes of entrapment include trauma, such as direct contusion to the nerve from fractures of the distal fibula, talus, calcaneus or base of the fifth metatarsal. Damage can occur during lateral ligamentous or peroneal tendon surgery. Other pathologies include traction injury with secondary fibrosis, tendinosis of the Achilles or peroneal tendons, gastrocnemius tear, and space-occupying lesions (Fig. 9). The sural nerve can also be damaged after small saphenous vein saphenectomy or after percutaneous suturing of the Achilles tendon [5, 52, 53]. The clinical presentation includes paraesthesia and/or pain along the lateral ankle and foot, which is exacerbated by inversion and plantar flexion of the foot, and chronic calf pain exacerbated by physical activity [5, 53]. On clinical examination, symptoms can be provoked by plantar flexion and inversion of the foot [2]. MRI may help identify the site of entrapment and the potential cause [2]. There is no muscular denervation oedema as the nerve is purely sensory [34].

### Obturator nerve

**Fig. 10:**
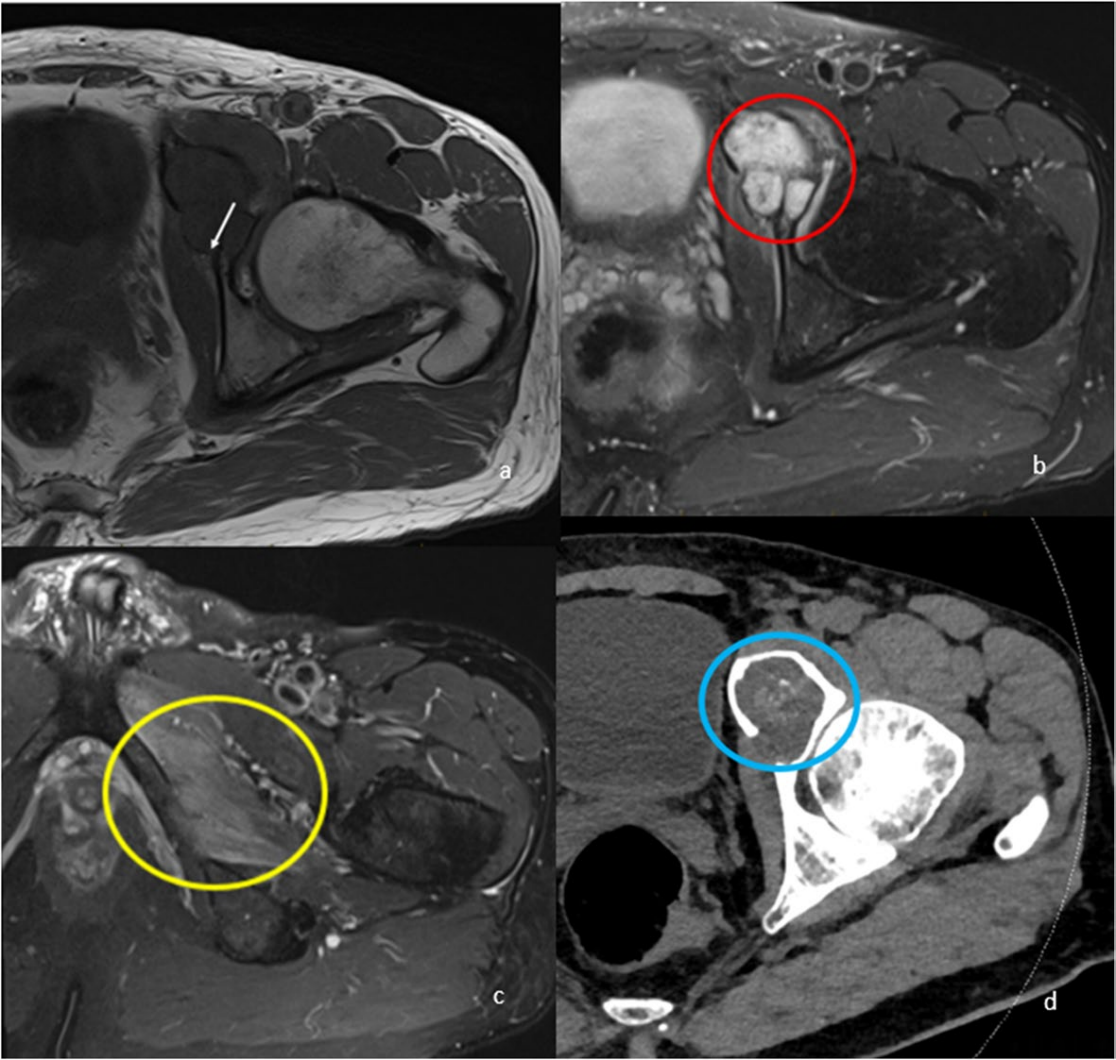
61-year-old male with chondrosarcoma in the left superior pubic ramus and anterior acetabulum. Axial T1 (**a**) and PDFS (**b**, **c**) sequences demonstrate the fluid-hyperintense intramedullary lesion with chondroid calcification (red circle). There is extraosseous extension into the left obturator foramen/canal, encroaching on the left obturator nerve (white arrow), resulting in nerve entrapment and secondary denervation oedema of the obturator externus, adductor brevis and magnus (yellow circle). CT reveals chondroid calcification of the tumour (blue circle) (**d**)

The obturator nerve arises from the anterior L2-L4 rami of the lumbosacral plexus and descends along the iliopectineal line before exiting the pelvis via the obturator canal at the superior aspect of the obturator foramen [3]. It provides sensory supply to the medial upper thigh and motor supply to the adductor brevis, adductor longus, adductor magnus, pectineus, gracilis and obturator externus. The nerve is most commonly entrapped in the obturator canal. Common causes of entrapment include pelvic trauma, periarticular cysts, perilabral cysts, hip surgery, prolonged labour and obturator herniation [3, 18, 34]. Rarely, entrapment can occur from mass lesions (Fig. 10) [3, 18, 34]. In athletes, neuropathy can develop from the formation of fibrous bands secondary to chronic adductor tendinopathy and osteitis pubis. Patients can present with groin or medial thigh pain associated with weakness of the adductor muscles [34, 54, 55]. They can also present with adductor muscle wasting and an abnormal wide gait [34, 54, 55]. MRI is the imaging modality of choice. Lesions causing mass effect on the neurovascular bundle can be directly visualised along with denervation changes in the adductor compartment muscles [55]. Alteration in size and signal of the entrapped nerve should be noted [34]. When the cause is not attributable to a lesion, muscle denervation changes may be the only imaging findings [18]. Treatment depends on the cause of entrapment and includes neurolysis and fascial and mass resection [2, 3].

### Lateral femoral cutaneous nerve

**Fig. 11:**
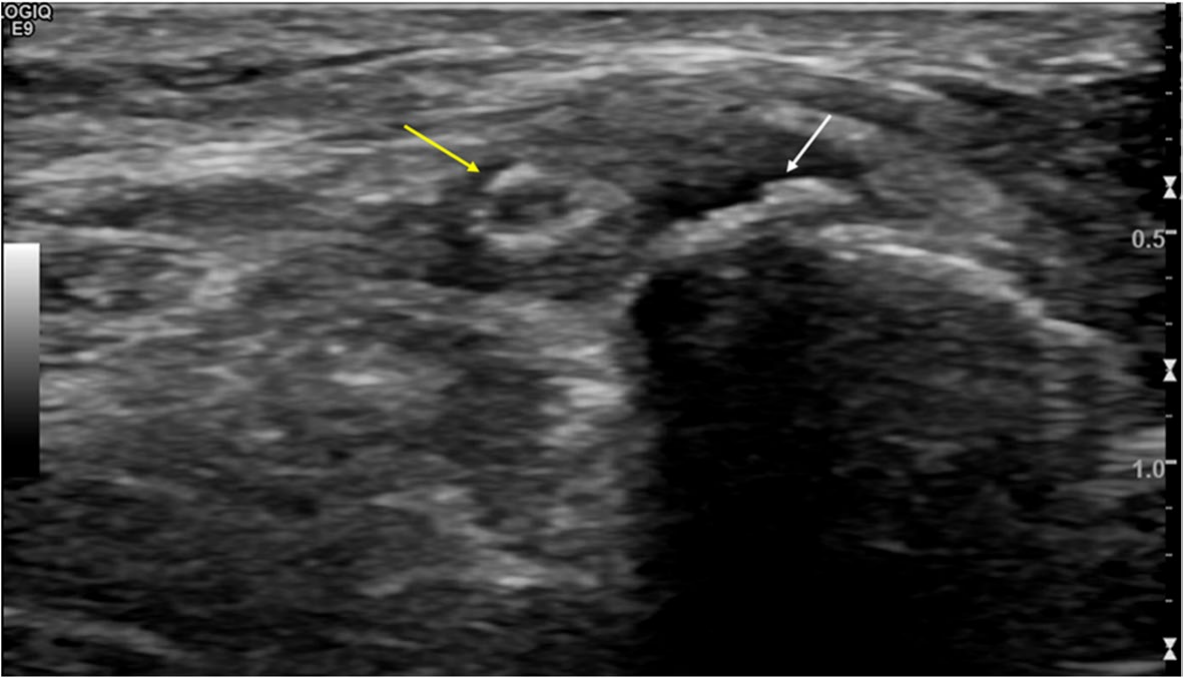
45-year-old male with enthesopathy at the anterior superior iliac spine (ASIS). Ultrasound demonstrates a traction spur arising from the ASIS (white arrow) with thickening of the inguinal ligament and lateral femoral cutaneous nerve (yellow arrow)

**Fig. 12:**
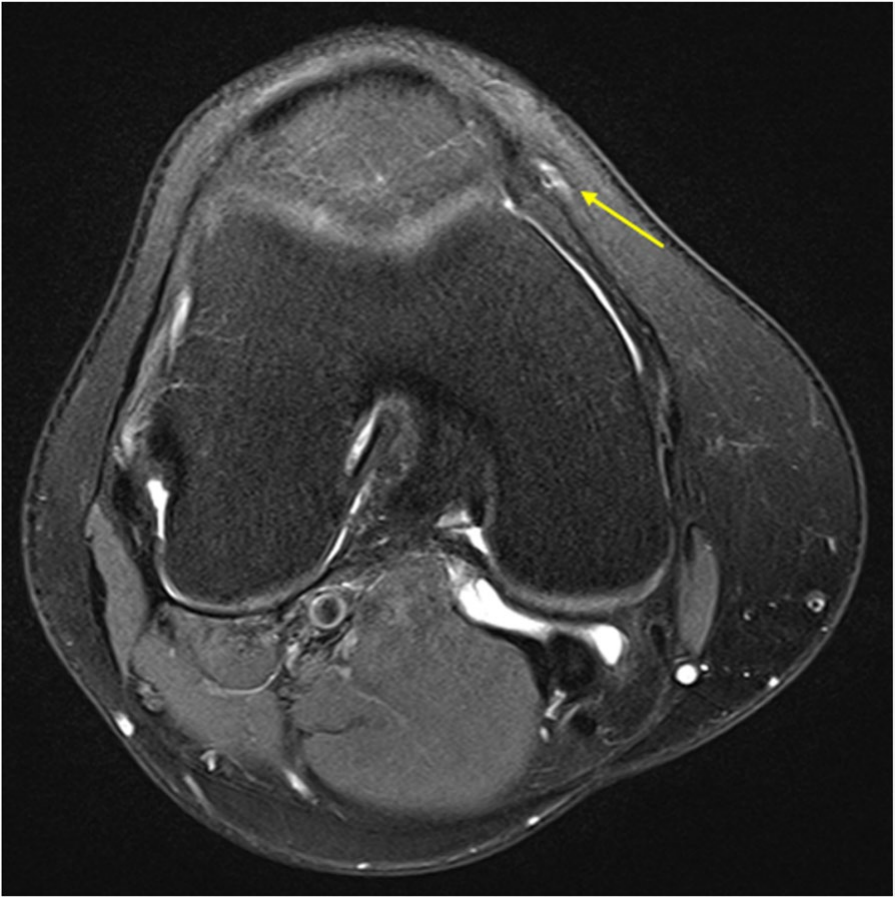
MRI of the left knee. Axial PDFS sequence. Fibrosis of the lower medial retinaculum. The infrapatellar branch of the saphenous nerve is oedematous (yellow arrow)

The lateral femoral cutaneous nerve is a sensory nerve arising from the dorsal divisions of L2 and L3. It emerges from the lumbar plexus on the lateral aspect of the psoas and descends obliquely on the anterior surface of the iliacus muscle. In most cases, it exits the pelvis deep to the inguinal ligament and medial to the anterior superior iliac spine [18]. It divides into an anterior branch which provides sensory supply to the anterolateral thigh, and a posterior branch which supplies sensation to the posterolateral thigh. Entrapment typically occurs as the nerve travels inferior to the inguinal ligament or as it pierces the fascia lata [34, 56]. There are multiple causes of entrapment, including avulsion fracture of the anterior superior iliac spine (ASIS), iatrogenic injury during pelvic fracture repair, inguinal or abdominal surgery, and pelvic and retroperitoneal tumours. Other contributing factors include discrepancy in limb length, diabetes, weight gain, pregnancy and external compression by belts or tight clothing [57–59]. The nerve is most commonly injured by mechanical compression at the level of the inguinal ligament [3]. Neuropathy of the lateral femoral cutaneous nerve has been termed meralgia paresthetica. Patients typically present with pain, and paraesthesia in the skin overlying the anterolateral aspect of the thigh [3]. The intrapelvic course of the nerve is not readily demonstrable on imaging. The extrapelvic course of the nerve can be visualised on MRI and ultrasound (Figs. 11 and 12). Nerve thickening and increased signal of the nerve can be seen [34, 60]. MRI can also identify lesions in the expected trajectory of the nerve [18]. Treatment options include removal of the offending cause of compression, such as through weight loss and wearing loose clothing [59]. Severe neuropathic pain can be treated with corticosteroid injections, radiofrequency ablation, or surgical intervention [43, 60].

### Femoral nerve

The femoral nerve receives fibres from the anterior rami of nerve roots L2, L3 and L4 and has both motor and sensory functions. Arising from the lumbar plexus, the femoral nerve descends through the psoas major muscle of the posterior abdominal wall. The femoral nerve gives branches to the iliacus and pectineus muscles. The nerve then passes underneath the inguinal ligament where it divides into anterior and posterior divisions. The anterior division gives anterior cutaneous branches, a branch to the sartorius muscle and a branch to the pectineus muscle. The posterior division of the femoral nerve gives branches to the quadriceps femoris. The femoral nerve supplies motor function to the muscles of the anterior thigh. This includes the hip flexors (pectineus, iliacus and sartorius) and knee extensors (quadriceps femoris). Sensory function of the femoral nerve arises from the anterior cutaneous branches and from the saphenous nerve. The anterior cutaneous branches innervate the skin of the anteromedial thigh [61]. The saphenous nerve is a branch of the posterior division of the femoral nerve which is particularly vulnerable to entrapment. It arises just distal to the inguinal ligament and travels into the adductor canal deep to the sartorius muscle. The nerve provides sensory innervation to the distal medial thigh, leg and foot [34, 62]. There are multiple causes of the saphenous neuropathy, including traumatic injury to the adductor canal, injury during knee surgery, compression from parameniscal cysts or ganglia, great saphenous vein saphenectomy and stretching injury due to posterolateral knee instability [22, 57]. Patients can present with the clinical symptoms of paraesthesia of the distal medial thigh, leg and foot [34, 62]. MRI findings include increase in size and T2 hyperintensity of the nerve (Fig. 12). Lesions with mass effect on the nerve can be visualised [34]. Lidocaine injection can be used for both diagnostic and therapeutic purposes [63].

## Conclusion

This article described and illustrated the imaging features of entrapment neuropathies of the lower limb and summarised the relevant anatomy. Ultrasound and MRI allow accurate direct evaluation of the nerves and indirect evaluation of neuropathy through assessment of the surrounding soft tissue structures.

## Data Availability

Not applicable.

## Abbreviations

CT: Computed tomography
DTI: Diffusion tensor imaging
MRI: Magnetic resonance imaging
MRN: Magnetic resonance neurography
PD: Proton density
PDFS: Proton density fat saturation
